# Coronavirus disease 2019 (COVID-19) pneumonia in a hemodialysis patient: A case report

**DOI:** 10.1097/MD.0000000000020956

**Published:** 2020-07-02

**Authors:** Luyan Huang, Yiru Wang, Lisheng Wang, Yongman Lv, Qingquan Liu

**Affiliations:** a Department of Anesthesiology, Hanyang Branch, Wuhan Hospital of Traditional Chinese Medicine; b Department of Nephrology; c Department of Health Management Center, Tongji Hospital, Tongji Medical College, Huazhong University of Science and Technology, Wuhan, China.

**Keywords:** 2019 novel coronavirus disease, coronavirus, hemodialysis, pneumonia, uremia

## Abstract

**Rationale:**

The 2019 novel coronavirus disease (COVID-19) causes a novel, atypical pneumonia that has brought huge public health challenges across the globe. There is limited data about patients with end-stage renal disease who also suffer from COVID-19. In this report, we discuss the case of a hemodialysis patient who developed COVID-19 pneumonia in the clinical course.

**Patient concerns:**

A 79-year-old man who had end-stage renal disease (ESRD) and was taking regular hemodialysis was admitted to hospital for a fever and dry cough. The patient, who also had cardiovascular disease, had no history of contact with COVID-2019 patients.

**Diagnosis:**

The patient was diagnosed with COVID-2019 by the reverse-transcriptase polymerase chain reaction (RT-PCR), and his pharyngeal swab for SARS-CoV-2 was positive.

**Intervention:**

The treatment was mainly supportive and the patient was intensively monitored. He was treated with oxygen, broad-spectrum antibiotics, antiviral drugs, and methylprednisolone. The patient took continuous renal replacement therapy (CRRT) every 2 days.

**Outcomes:**

After 19 days, an RT-PCR assay for SARS-CoV-2 was negative, and computed tomography (CT) of the patient's thorax indicated that the pulmonary inflammatory exudation was absorbed and pulmonary infection improved significantly. He was discharged on day 29 after recovering from COVID-2019 pneumonia.

**Lessons:**

The courses of disease and treatment options for this individual were significantly more complicated than those for ordinary patients. Therefore, it was necessary to monitor the condition of the patient closely and to protect the dialysis unit staff from being infected. Compared with other severe COVID-2019 cases, this patient recovered more quickly following treatment, which was likely due to the removal of inflammatory mediators by CRRT. This implies that blood purification might be an important option for hemodialysis patients with COVID-19.

## 1 Introduction

In December 2019, an outbreak of novel coronavirus (COVID-2019) was first reported and confirmed in Wuhan, China. The main clinical manifestations of the virus are fever, weakness, cough, and severe respiratory difficulties or even respiratory failure.^[1,2]^ COVID-2019 is highly contagious by close contact, and outbreaks and case clusters have been reported in the literature. The sequence homology of COVID-2019 and SARS is 79%, and mechanisms of action of the viruses are similar.^[3]^ Studies have shown that the clinical manifestations of patients with SARS who are on dialysis are not typical and can be easily overlooked until they progress.^[4]^ COVID-2019 infection can occur in both healthy individuals and those with chronic illness such as end-stage renal failure. An understanding of whether the clinical processes and infection outcomes of patients with kidney disease in COVID-2019 infection differ from those of other infected patients is crucial. However, the clinical information on renal dialysis patients with COVID-2019 is insufficient. We describe here the case of one end-stage renal disease (ESRD) patient who received regular hemodialysis and was infected with SARS-CoV2.

## 2 Case presentation

A 79-year-old man, who had end-stage renal disease and received regular hemodialysis 3 times a week for no >2 months, was admitted to the Tongji Hospital on January 25, 2020 because of a fever of 37.7 °C and a dry cough for 2 days. He was also weak and had malaise and anorexia. He had no history of contact with COVID-2019 patients. The patient had other comorbid diseases including hypertension for 30 years, gout for 2 years, and coronary heart disease for 1 month, the latter of which required coronary stenting on December 19, 2019. He received right nephrectomy for severe right kidney hydronephrosis 35 years ago. Due to his clinical symptoms, he was diagnosed with novel coronavirus (COVID-2019). The laboratory data diagnosis of COVID-19 pneumonia was based on the New Coronavirus Pneumonia Prevention and Control Program (4th edition) published by the National Health Commission of China (http://www.gov.cn/zhengce/zhengceku/).

### 2.1 Clinical findings

After admission, his laboratory data revealed that the pharyngeal swab nucleic acid test for COVID-2019 was positive. A routine blood test showed that his peripheral white cell count was 6.39 × 10^9^/L (normal range [NR], 3.5–9.5 × 10^9^/L), lymphopaenia count was 0.92 × 10^9^/L (NR, 1.1–3.2 × 10^9^/L), neutrophils count was 4.93 × 10^9^/L (NR, 1.8–6.3 × 10^9^/L), and platelet count was 173 × 10^9^/L (NR, 125–350 × 10^9^/L). His elevated erythrocyte sedimentation rate (ESR) and hypersensitive C-reactive protein (hsCRP) level were 75 mm/H and 119.9 mg/L, respectively. His serum creatinine was 776 mmol/L, blood urea nitrogen was 26.93 mmol/L, and lactate dehydrogenase was 229 U/L (NR, 135–225 U/L), but his liver enzymes, creatinine kinase, and alkaline phosphokinase were within normal ranges. His NT-proBNP was higher than 70,000 pg/mL, and his hypersensitive cardiac troponin I was 85.4 pg/mL. The cytokines levels were as follows: interleukin-2 receptor 2575 U/mL (NR, 223–710 U/mL), interleukin-6 88 pg/mL (NR, <7.0 pg/mL), interleukin-10 36.7 pg/mL (NR, <9.1 pg/mL), and tumor necrosis factor α 28.2 pg/mL (NR, <8.1 pg/mL). Other type 1 cytokines, including interleukin-1β and interleukin-8, were within normal ranges.

### 2.2 Treatment and outcomes

**Figure 1:**
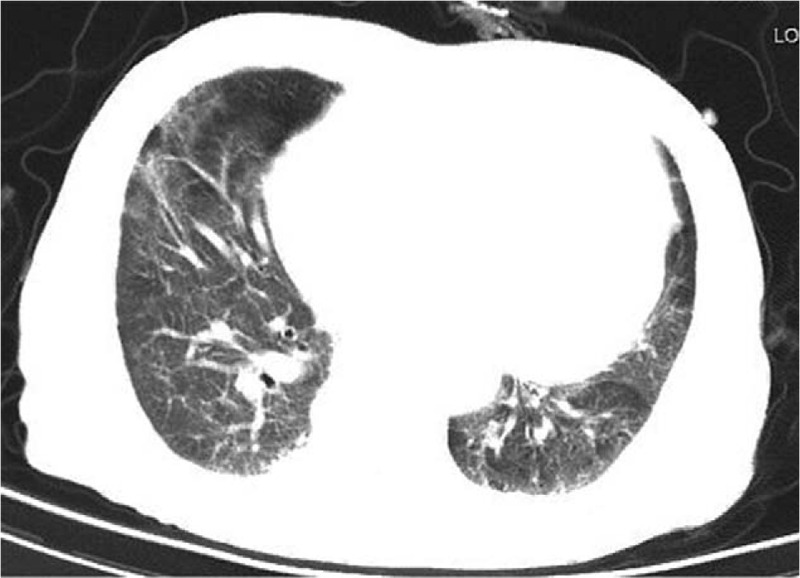
High resolution computed tomography of thorax at initial presentation. There is diffuse ground glass opacification, bilateral patchy consolidation, and fibrosis, features compatible with recovery phase of pneumonia.

The patient was treated with intravenous moxifloxacin (broad-spectrum antibiotic) at 400 mg and ganciclovir at 1.25 mg/kg, once daily, and atomization inhalation of 500wu recombinant human interferon α2b every 12 hours. He was also treated with bedside continuous renal replacement therapy (CRRT) for 8 hours every 2 days, and the dialysis model of continuous veno-venous hemodiafiltration (CVVHDF) or continuous veno-venous hemofiltration (CVVH) was selected according to the patient's treatment needs. (Blood flow, 150–200 mL/min; ultrafiltration rate, 150–200 mL/h). His highest temperature was 38.4 °C, and his fever began to subside on day 3. However, on day 2, he began to have difficulty breathing, and his oxygen saturation was 89%. He was given a nasal catheter for oxygen, followed by ambroxol hydrochloride at 300 mg and methylprednisolone at 40 mg every 24 hours. On day 3, as his dyspnoea worsened, he was given a high flow of oxygen (40 L/min) through the nose. On day 8, his dyspnoea improved significantly; his oxygen saturation was above 95% when setting the flow of oxygen at 3 L/min. The patient received daily prednisone at 20 mg starting on day 11. On day 15, the levels of interleukin-6 and tumor necrosis factor α had decreased to 4.99 pg/mL, 12.4 pg/mL, respectively, and the hsCRP level was 8.4 mg/L (Table 1). The RT-PCR assay for COVID-2019 was negative on day 19, and a high-resolution thorax CT on day 22 showed diffuse ground glass opacification, bilateral patchy consolidation, and fibrosis, which were features of the recovery phase of acute pneumonia (Fig. 1). He was discharged on day 29 after recovering from COVID-2019 pneumonia.

**Table 1:**
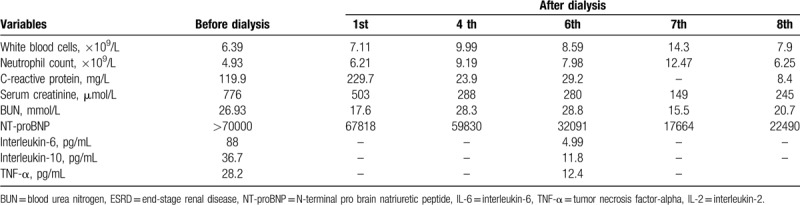
Dynamic changes of laboratory characteristics before and after dialysis of the ESRD patient with COVID-19.

### 2.3 Hemodialysis management

The patient was admitted to the infection ward and received regular blood purification at the bedside. To stay protected, each staff member wore a waterproof disposable gown, cap, gloves, face shield, and N95 facemask, as recommended by the WHO. Patients who undergo continuous RRT may either take continuous veno-venous hemofiltration (pre-filter fluid replacement, CVVH) or continuous veno-venous hemodiafiltration (post-filter fluid replacement, CVVHDF) with AQUARIUS^TM^ (NIKKISO, Shanghai, China). Fresenius polysulfone dialyzer AV600s (Fresenius Medical Care, Bad Homburg, Germany) were applied without reuse. Spent dialysate was drained directly to the ward sewer, which was connected to the main sewage drain by a U-trap. The dialysis machine was disinfected by 75% ethanol, kept in the infection ward, and only used for patients who were diagnosed with COVID-19 pneumonia and required dialysis.

The patient provided informed consent for the publication of his clinical data. Ethical approval was not necessary because this is a retrospective case report. The presented data are anonymized, and risk of identification is minimal.

## 3 Discussion

In this report, we describe the clinical course of a hemodialysis patient with COVID-19 pneumonia. CRRT not only improved the patient's levels of blood urea nitrogen, serum creatinine, and potassium, but also reduced the levels of inflammatory mediators to some extent.^[5]^ The initial clinical presentation of the patient was similar to that of other COVID-19 pneumonia patients. COVID-2019 is a novel virus discovered in Wuhan, China that mainly presents with respiratory symptoms, such as fever, dry cough, and, in severe cases, breathing difficulties, acute respiratory distress syndrome (ARDS), and even respiratory failure.^[2,6]^ Its main route of transmission is close contact. The incidence of COVID-2019 infection in men and women is 0.31 per 100,000 and 0.27 per 100,000, and the mortality rate is approximately 3.06% in China.^[7]^ COVID-2019, SARS-CoV, and MERS-CoV are pathogenic human coronaviruses^[3]^ that cause a wide range of clinical manifestations in humans, with a large proportion of patients developing short-term, moderate clinical disease, and a small but significant number of patients experiencing severe disease characterized by ARDS.^[8,9]^ Despite years of research, specific factors leading to the high morbidity and mortality rates of pathogenic human coronaviruses have not been fully understood.

The patient in our study had a history of several comorbid diseases including coronary heart disease (CHD). Specifically, he had uremia and received coronary stenting for CHD only 1 month prior to his hospitalization. The abnormal hypersensitive cardiac troponin I level and high level of NT-proBNP suggest the patient had myocardial ischemia and heart failure. Given that these changes, which are highly lethal, should be taken into consideration when setting the parameters (e.g., blood flow and ultrafiltration rate) for blood purification therapy in this patient. Proper ultrafiltration rates, blood flow rate, and dialysate flow rate could keep hemodynamic stability of CRRT and then contribute to be cardio-protective.^[10]^ Therefore, during the process of CRRT, the dialysate flow rate was 1000 to 1500 mL/h and blood flow rate was between 150 and 200 mL/min.

Cytokines have an established, important role in the immunopathology of viral infections. The expression of cytokines, like IL-6 and TNF-α, was shown to be up-regulated in SARS-CoV-infected dendritic cells, and the excessive secretion of IL-6 may serve as an immunopathological process that causes lung injury in SARS.^[11–13]^ The patient in our study had high levels of IL-6, TNF-α, and IL-2 receptor. Hence, when we compiled blood purification protocols, the need to remove both toxins and inflammatory cytokines was considered. Hemodialysis can remove excess inflammatory mediators and restore immune balance.^[14]^ Therefore, CVVHDF was the preferred treatment model to remove both toxins and inflammatory cytokines from our patient. After 8 times of blood purification, the levels of inflammatory mediators in the patient decreased significantly, and his condition gradually improved. We speculate that his stable recovery required the blood purification therapy, and suggest that hemodialysis can benefit patients with severe COVID-2019 pneumonia. Similarly, in a recently study of 5 maintenance hemodialysis patients with COVID-19 disease, none of the patients died or developed severe complications such as acute respiratory distress syndrome, shock, or multiple organ dysfunction.^[7]^ As hemodialysis can remove the concentration of drugs in the blood, and patient's condition gradually recovered steadily. So the treatment time of blood purification was set 8 hours each time, in this case, longer non-dialysis periods are required for drug infusion.

If the level of inflammatory mediators in the patient's body gradually rises or the condition gradually worsens, the treatment time for blood purification should be prolonged, and the parameters of blood flow and ultrafiltration should be lowered again to ensure more stable circulation in body.

The diameter of coronavirus is approximately 50 to 120 nm,^[15]^ but the diameter of the dialyzer membrane is approximately 3 to 10 nm. Therefore, coronavirus cannot pass through the conventional low-flux polysulfone membrane.^[16]^ We speculate that it was safe to drain the spent dialysate through ordinary sewer but did not test the dialysate for the presence of COVID-2019.

In summary, we described a hemodialysis patient who suffered from COVID-2019. With appropriate infection control measures and symptomatic supportive treatment, staff members taking care of the patient were not infected. Blood purification for the removal of inflammatory cytokines may serve as an effective treatment option for hemodialysis patients with viral pneumonia.

## Acknowledgments

The authors thank AiMi Academic Services (www.aimieditor.com) for English language editing and review services.

## Author contributions

LYH and LSW did the the data collection, LYH and YRW literature review, analyzed the data, and wrote the first draft of the manuscript. QQL and YML participated in the revision of the manuscript. QQL approved the version.
